# Genetic variation and phylogeographic structure of *Spodoptera exigua* in western China based on mitochondrial DNA and microsatellite markers

**DOI:** 10.1371/journal.pone.0233133

**Published:** 2020-05-14

**Authors:** Xing-Ya Wang, Ming-Ming Wang, Chen Chen, Xiao-Qi Wang

**Affiliations:** College of Plant Protection, Shenyang Agricultural University, Shenyang, Liaoning, China; CMAVE, USDA-ARS, UNITED STATES

## Abstract

The beet armyworm, *Spodoptera exigua*, is a significant agricultural pest of numerous crops and has caused serious economic losses in China. To effectively control this pest, we analyzed its genetic variation, population genetic structure and demographic history. We used mitochondrial DNA (mtDNA) fragments of the *cytochrome oxidase subunit I* (*COI*) and eight nuclear microsatellite loci to investigate genetic diversity and population genetic structure of *S*. *exigua* populations at 14 sampling sites in western China. Both mtDNA and microsatellite data indicated low levels of genetic diversity among all populations. A moderate genetic differentiation among some *S*. *exigua* populations was detected. Neighbor-joining dendrograms, STRUCTURE, and principal coordinate analysis (PCoA) revealed two genetically distinct groups: the KEL group and the remaining population group. Isolation by distance (IBD) results showed a weak significant correlation between geographic distance and genetic differentiation. Haplotype networks, neutrality testing, and mismatch distribution analysis indicated that the beet armyworm experienced a recent rapid expansion without a recent genetic bottleneck in western China. Thus, the results of this population genetic study can help with the development of strategies for managing this highly migratory pest.

## Introduction

The beet armyworm, *Spodoptera exigua* (Lepidoptera: Noctuidae), is a significant polyphagous pest on vegetables, maize, cotton, soybeans, and ornamental plants [1,2]. Generally, *S*. *exigu*a larvae feed on host leaves, decreasing crop yields and leading to the death of plants. Originating from South Asia, this species is already widely distributed in the tropical and temperate regions of Europe, Africa, North America, and Asia [3]. In China, *S*. *exigua* was first recorded in Beijing in the 1890s. It has a wide distribution in the main crop-producing areas of China and has caused severe economic losses in recent years. For example, *S*. *exigua* has spread to some provinces in North and East China and has infested a total area of over 2.7 million hectares [4]. This pest especially damages welsh onions in Northern China and has infested more than 8000 hectares in Tianjin, reducing the annual welsh onion production by 30% [5,6].

*Spodoptera exigua* was a polyphagous insect and has high fecundity and long-distance flight capabilities [1,7]. In general, eggs of this species are deposited on the undersides of leaves, the newly hatched larvae feed gregariously on the back of the leaves, the third instar begins to feed solitarily, and the fourth instar begins to eat a large number of leaves, petals, and pods. Pupae mainly live through the winter in the soil, and no overwintering occurs in South China. They can reproduce all year round, and any diapause behavior has not been reported [8]. Chemical pesticides are still a predominantly applied type of pest control. Due to the long-term use of chemical insecticides, *S*. *exigua* rapidly developed resistance to many of them, such as chlorinated hydrocarbons and carbamates [9,10]. Therefore, effectively controlling this pest is difficult.

The genetic variation and population genetic structure of a species can be affected by many factors, such as climate change, ecological factors, natural barriers, migration behavior, and human activities [11–14]. Nowadays, a variety of molecular markers have been used to infer the species’ biogeography and evolutionary history [15]. Of these markers, due to its moderate evolutionary rate and a clear evolutionary pattern, the *cytochrome oxidase subunit I* (*COI*) gene is suitable for reconstructing species phylogenies [16,17]. Due to the characteristics of high codominance and high polymorphism, microsatellite markers have been applied extensively in population genetics studies [18,19]. These studies provide valuable information for understanding the dispersal pattern and the causes of outbreaks of pest species. However, information research on the genetic variability and genetic structure of *S*. *exigua* on large spatial scales is lacking, and only few studies being conducted to date [17,20–22]. Due to the use of small sample sizes and the limited number of populations, accurate assessments of the genetic diversity and population genetic structure of this pest on a large scale with respect to regions in western China have not been provided.

In the present study, we investigated the population genetics of *S*. *exigua* in western China. Both the mitochondrial DNA (mtDNA) *COI* gene and microsatellite loci were used to infer the genetic variation, genetic differentiation, population genetic structure, and demographic history of 14 *S*. *exigua* populations in western China. We discussed the management strategies for this species. This study aims to recognize the population genetics of this moth and provide a useful theoretical basis for developing effective management strategies for this pest.

## Materials and methods

### Sample collection and DNA extraction

**Fig 1:**
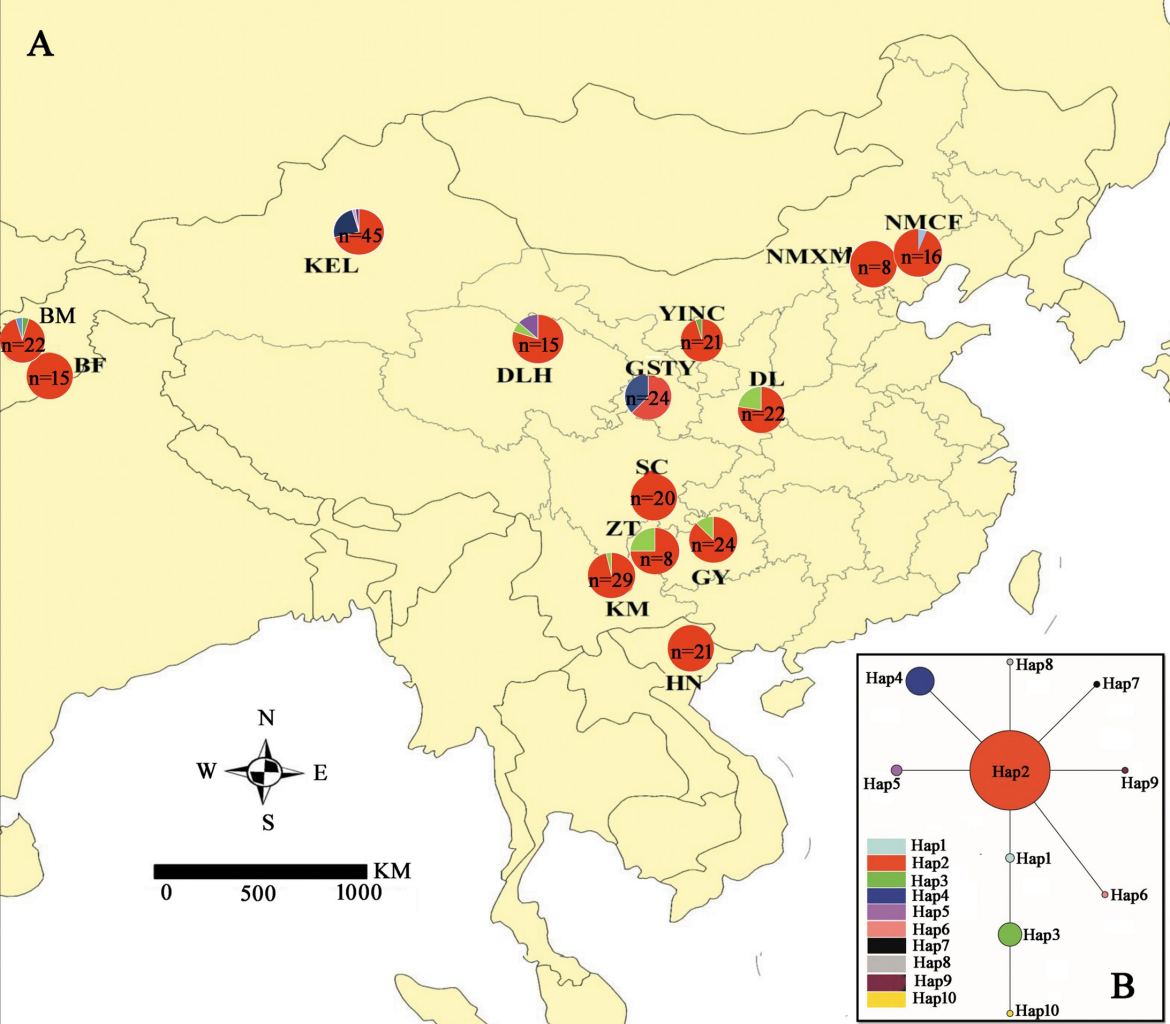
Sampling locations and haplotype frequencies in 14 *Spodoptera exigua* populations in China. (A) Mitochondrial *cytochrome oxidase subunit I* (*COI*) haplotype frequencies. (B) Mitochondrial *COI* haplotype network. Each line between circles represents one mutational event. The area of a circle is proportional to the number of observed individuals. For population abbreviations, see S1 Table. The figure is modified from the graphic of Song et al. [23]. *n* in the pie chart represents the sample size of each population.

All individuals of *S*. *exigua* in the 14 locations were collected by using sex pheromone traps from June to October from 2012 to 2018, including 11 populations collected in western China, 2 populations collected in Multan, Pakistan, and 1 population collected in Hanoi, Vietnam. Details of the locations of populations and the numbers of samples are provided in Fig 1 and S1 Table. Samples were collected on private land with permission of the land owners, and none of the field surveys in the present study involved endangered or protected species. All the *S*. *exigua* samples were preserved in 95% ethanol at −20°C and deposited in the Plant Protection College, Shenyang Agricultural University, Shenyang, China. Genomic DNA was extracted from individual samples using Qiagen’s DNEasy Blood & Tissue kit (Qiagen, Valencia, CA, USA) according to the manufacturer’s protocols.

### Amplification of mitochondrial DNA and sequencing

A fragment of the *COI* mitochondrial genes was amplified by PCR using the primers LCO1490 (5′-GGTCAACAAATCATAAAGATATTGG-3′) and HCO 2198 (5′-TAAACTTCAGGGTGACCAAAAAATCA-3′) [24]. Each PCR mixture contained 1.25 units of EasyTaq DNA polymerase, 1× Easy Taq^®^buffer (including 2 mM MgCl_2_; Transgen Biotech Co., Ltd, Beijing, China), 0.5 μM of each primer, 1 μL of DNA template, and 2.5 mM dNTP mixture. Conditions for PCR amplification were as follows: 94°C for 5 min; 35 cycles at 94°C for 30 s, 52°C for 30 s, and 72°C for 45 s; and a final extension at 72°C for 5 min. Amplification products were purified and then sequenced by Sangon Biotech (Shanghai) Co., Ltd. Chromatograms, including sense and antisense, were edited and assembled using DNASTAR 5.0 (DNASTAR, Madison, WI, USA) to obtain a single consensus sequence. Microsatellite alleles were analyzed using GeneMapper 4.0 software (Applied Biosystems).

### Microsatellite amplification and genotyping

In this study, individuals were genotyped using eight microsatellite loci, and technical details were provided by Kim et al. [25]. These microsatellite loci were assigned unique fluorophores for the fluorescent tagging of DNA in a PCR. For these isolated microsatellites, each PCR mixture contained 1.0 units of EasyTaq DNA polymerase, 2.5 mM dNTP mixture, 1× Easy Taq^®^buffer (including 2 mM MgCl_2_; Transgen Biotech Co., Ltd, Beijing, China), 0.5 μL of DNA template, and 0.4 μM of each primer, which were labeled with fluorochromes HEX or FAM (Sangon Biotech, Shanghai, China). Conditions for PCR amplification were as follows: 94°C for 4 min; 30 cycles at 94°C for 30 s, 58°C for 30 s, and 72°C for 30 s; and a final extension at 72°C for 5 min. Following amplification, the products were visualized at Sangon Biotech Co., Ltd. (Shanghai, China) using an ABI 3730XL automated sequencer (Applied Biosystems, Foster City, CA, USA).

### Data analyses

#### Mitochondrial data

The mtDNA *COI* sequences were aligned manually using the CLUSTAL X 1.81 program [26] and were subsequently checked manually. We used MEGA 5.2 to analyze the sequence variation information and genetic distance between DNA sequences based on Kimura’s two-parameter method [27]. For each population, haplotype diversity (*π*) and nucleotide diversity (*h*) were estimated using DnaSP 4.0 software [28]. The median-joining network of the mtDNA *COI* haplotypes was conducted in NETWORK 2.0 [29]. We calculated population genetic differentiation (*F*_ST_) statistics between populations using Arlequin 3.0 [30]. Analysis of molecular variance (AMOVA) based on *F*_ST_ and haplotype frequencies were completed using Arlequin 3.0. Isolation by distance (IBD) was determined by testing the correlation between geographical distance and pairwise *F*_ST_*/*1–*F*_ST_ using the Mantel test with Arlequin 3.0 [30]. Demographic history changes were analyzed for *S*. *exigua* using two neutrality tests, Tajima’s *D* [31] and Fu’s *F*_S_ [32], across the 14 geographic populations. Finally, mismatch distributions were calculated between the observed and expected mismatch distributions by Arlequin 3.0 [29].

#### Microsatellite data

The software Micro-Checker 2.2.3 [33] was used to test for errors and null alleles. Genotypic linkage disequilibrium (LD) and Hardy–Weinberg equilibrium (HWE) were computed using GenePop 3.4 [34]. Corrections for multiple tests were performed via Bonferroni corrections. Genetic diversity indexes, such as the mean number of alleles (*Na*), the effective number of alleles (*Ne*), Shannon's information index (*I*), observed heterozygosity (*H*_O_), expected heterozygosity (*He*), and unbiased expected heterozygosity (*uH*e) were determined using GenAlEx 6.41 [35]. Allelic richness (*A*_*R*_), the fixation index (*F*_ST_), and the inbreeding coefficient (*F*_IS_) were calculated with FSTAT 2.9.3.2 [36]. Polymorphism information content (*PIC*) was calculated in CERVUS 2.0 [37]. The levels of genetic differentiation between pairs of populations were also estimated in Arlequin 3.0 [30].

We used POPTREE 2 to construct an unrooted phylogenetic tree by the neighbor-joining method with 1,000 bootstrap replicates [38]. The software STRUCTURE 2.3 [39] was used to identify clusters of genetically similar populations based on the Bayesian approach. Markov chain Monte Carlo (MCMC) values were set for a burn-in period of 30,000 and a run length of 105 iterations under an admixture model with correlated allele frequencies within populations. To identify the optimal number of groups (*K*), measures of change in the likelihood function (*Δ*K) and *F*_ST_ (*ΔF*_ST_) were calculated using the package CORRSIEVE [40], and the plateau criterion was applied [41]. The software CLUMPP 1.1 [42] was used to conduct model averaging of individual ancestry coefficients across 10 independent runs. Next, clusters were visualized using DISTRUCT 1.1 [43]. PCoA, based on the covariance of the genetic distance matrix, was implemented in GenAlex 6.41 [35]. The significance of the hierarchical partitioning of the genetic structure among the geographic groups was tested using an AMOVA, which was conducted in Arlequin 3.0 [30]. We used the program Bottleneck 1.2.02 to investigate whether any of the populations experienced genetic bottlenecks [44]. We performed the test using the infinite allele (IAM), strict stepwise mutation model (SMM), and two-phase (TPM) models of microsatellite evolution. We assessed the significance of the tests using Wilcoxon’s test, which is the most appropriate test in cases where fewer than 20 microsatellite loci are used [45]. Asymmetric pairwise measures of a recent gene flow were estimated using a genetic approach, assignment tests, and detection first-generation migrants for each population implemented in GENECLASS 2.0 software. This software was also used to identify first-generation migrant individuals of *S*. *exigua* using a partial Bayesian method [46].

## Results

### Genetic diversity

In this study, 312 individuals were collected from 14 populations in western China and genotyped with eight microsatellite loci. The null allele frequency per locus was mostly less than 0.1 (S2 Table). The Hardy–Weinberg expectations (HWE) test showed that 57 of the 112 locus–population combinations had significantly deviated, and no significant linkage disequilibrium was found. The average *F*_ST_, which was not used, and the excluding null alleles (ENAs) per the locus correction method, was 0.184 and 0.171, respectively, and these *F*_ST_ estimations did not differ significantly (*P* = 0.462). Therefore, the presence of null alleles did not influence *F*_ST_ estimation (S3 Table). Modern genetic variation among eight microsatellite loci of *S*. *exigua* in western China was found (S4 Table). Overall, the middle level of genetic diversity of all of the populations in the sampled regions was obtained, and the mean observed heterozygosity was equal to the mean expected heterozygosity (S5 Table). The estimates of microsatellite genetic variation differed among these populations. For example, the observed number of alleles (*Na*) across microsatellite loci ranged from 4.250 in Xilengrad (NMXM) to 8.875 in Kunming (KM). The effective number of alleles (*Ne*) ranged from 2.908 in Haikou (DLH) to 4.166 in Guiyang (GY). Shannon's information index (*I*) across loci ranged from 1.130 to 1.545. The inbreeding coefficients (*F*_IS_) of the eight microsatellite loci in all 14 populations of *S*. *exigua* were nearly zero (*F*_IS_ *=* 0.131). Kunming (KM) population had the highest genetic diversity, whereas the DLH population had the lowest genetic diversity.

A 576 bp fragment of the mtDNA *COI* gene sequence was sequenced for 291 *S*. *exigua* individuals (deposited in GenBank under accession nos. KY069907–KY069921). Of the 576 total characters, 566 were conserved sites and 10 were variable sites. We observed 10 haplotypes in 291 individuals. The average number of haplotypes in each population was 2.286 ± 0.914 and ranged from 1 to 4, and the Korla (KEL) population had the highest number of haplotypes. Hap 2 was the most common haplotype, present in all populations, and was shared by 247 individuals. Hap 4 was the second most frequent haplotype, being shared by 20 individuals from two populations. Genetic diversity indices haplotype diversity (*h*) and nucleotide diversity (π) are shown in S6 Table. On average, *h* and *π* were 0.273 and 0.00069, respectively. The highest genetic diversity (*h* = 0.489, *π* = 0.00085) was detected in Taiyuan (GSTY). In contrast, the genetic diversity value of Xilengrad (NMXM) and Hanoi (HN) was zero because no variable sites were found (S6 Table).

### Median-joining network and genetic differentiation

These 10 mtDNA *COI* haplotypes were from 14 locations, which covered more than 4,000 km. These results indicated the random distribution of mtDNA *COI* haplotypes in *S*. *exigua*, which may reflect a high level of gene flow in this species. Furthermore, the median-joining network of mtDNA *COI* haplotypes indicated no apparent geographical clustering of haplotypes (Fig 1). A star-shaped network suggested that many haplotypes differed from Hap 2 by only one or two mutations, which suggested that *S*. *exigua* experienced a population expansion event.

A global *F*_ST_ value of 0.217 was obtained based on the microsatellite data from all populations. The pairwise *F*_ST_ values between populations ranged from −0.053 to 0.484, and the exact tests detected that 68 of the 91 pairwise comparisons differed significantly (Table 1). Overall, a moderate level of genetic differentiation among populations was determined. We found significant differentiation between most populations, such as between Korla (KEL), Delingha (DLH), Kunming (KM), Chengdu (SC), Multan (BM), Faisalabad (BF), and Hanoi (HN). We also found significant differentiation between Yinchuan (YINC), Guiyang (GY), and other populations. Based on the mitochondrial DNA data, the pairwise *F*_ST_ values computed from ranged from –0.20 to 0.94, with an average *F*_ST_ of 0.109 (Table 1). We identified a significant genetic differentiation in 24 of the 105 comparisons (*P* < 0.05). Although a significant difference existed between most populations including Korla (KEL) and Lanzhou (GSTY), we found no significant differentiation among the other remaining populations.

**Table 1:** The pairwise *F*_ST_ among 14 population of *Spodoptera exigua* in western region based on eight microsatellite loci (below diagonal) and mtDNA *COI* gene sequences (above diagonal).

Pop.	NMCF	NMXM	DL	GSTY	YINC	KEL	DLH	ZT	KM	GY	SC	BM	BF	HN
NMCF	0.000	−0.05	0.099	**0.268**	−0.041	**0.103**	0.006	0.152	−0.032	0.008	0.001	−0.036	−0.031	0.021
NMXM	0.007	0.000	0.093	**0.226**	−0.057	**0.061**	−0.018	0.143	**−0.062**	0.006	−0.056	−0.04	−0.048	**0.000**
DL	**0.037**	−0.001	0.000	0.264	0.083	0.199	0.049	−0.092	0.118	−0.008	0.164	0.05	0.036	0.190
GSTY	**0.055**	0.035	0.010	0.000	**0.27**	0.005	**0.22**	**0.275**	**0.282**	**0.243**	**0.291**	**0.24**	**0.221**	**0.336**
YINC	**0.030**	0.009	0.022	**0.024**	0.000	**0.111**	0.000	0.122	−0.035	−0.009	−0.001	−0.033	−0.04	0.002
KEL	**0.343**	**0.414**	**0.428**	**0.452**	**0.400**	0.000	**0.104**	**0.206**	**0.12**	**0.135**	**0.114**	**0.112**	**0.099**	**0.125**
DLH	**0.150**	**0.207**	**0.277**	**0.305**	**0.264**	0.411	0.000	0.038	0.017	−0.003	0.045	−0.006	−0.018	0.069
ZT	0.009	0.207	0.020	0.021	0.014	**0.373**	**0.250**	0.000	0.166	−0.027	0.249	0.056	0.033	0.332
KM	0.022	0.039	**0.104**	**0.119**	**0.086**	**0.323**	**0.119**	**0.047**	0.000	0.017	−0.005	−0.016	−0.023	−0.01
GY	−0.008	−0.020	0.003	0.021	0.010	**0.380**	**0.197**	**0.005**	**0.053**	0.000	0.064	−0.021	−0.029	0.08
SC	**0.112**	**0.116**	**0.063**	**0.027**	**0.077**	**0.484**	**0.387**	**0.079**	**0.196**	**0.078**	0.000	0.016	0.011	0.005
BM	**0.118**	**0.176**	**0.245**	**0.271**	**0.230**	**0.389**	−0.005	**0.212**	**0.110**	**0.178**	**0.341**	0.000	−0.037	0.024
BF	**0.100**	**0.258**	**0.269**	**0.294**	**0.250**	**0.357**	−0.010	**0.262**	**0.097**	**0.182**	**0.373**	−0.053	0.000	0.027
HN	**0.056**	**0.044**	**0.051**	**0.031**	**0.036**	**0.422**	**0.301**	0.017	**0.115**	**0.025**	**0.077**	**0.274**	**0.282**	0.000

Bold indicates significant values after Bonferroni correction (*P* = 0.05).

### Population genetic structure

#### POPTREE analysis based on microsatellite data

**Fig 2:**
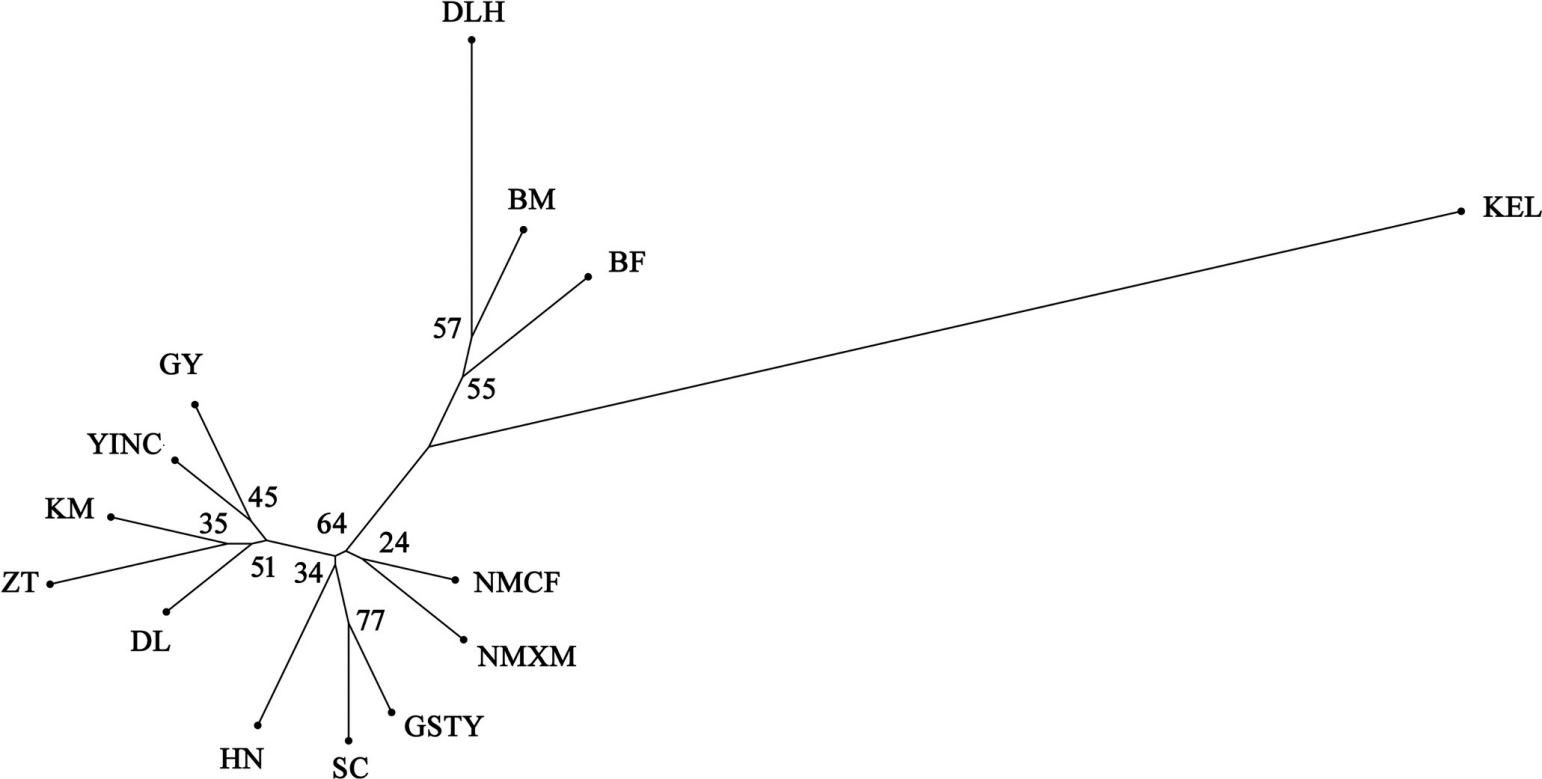
Unrooted neighbor-joining phylogenetic tree based on microsatellite data from 14 *S*. *exigua* populations in western China. The numbers beside the nodes are bootstrap values.

Based on microsatellite data, the unrooted neighbor-joining tree, including the 14 *S*. *exigua* populations defined two major clades (Fig 2). One clade corresponded to the Korla (KEL) population, which originated from Xinjiang, and the second clade was composed of the remaining 13 populations in western China. Due to the absence of a strong phylogeographic structure in the latter clade, frequent gene flow in the vast western region of China is suggested.

#### Bayesian clustering

**Fig 3:**
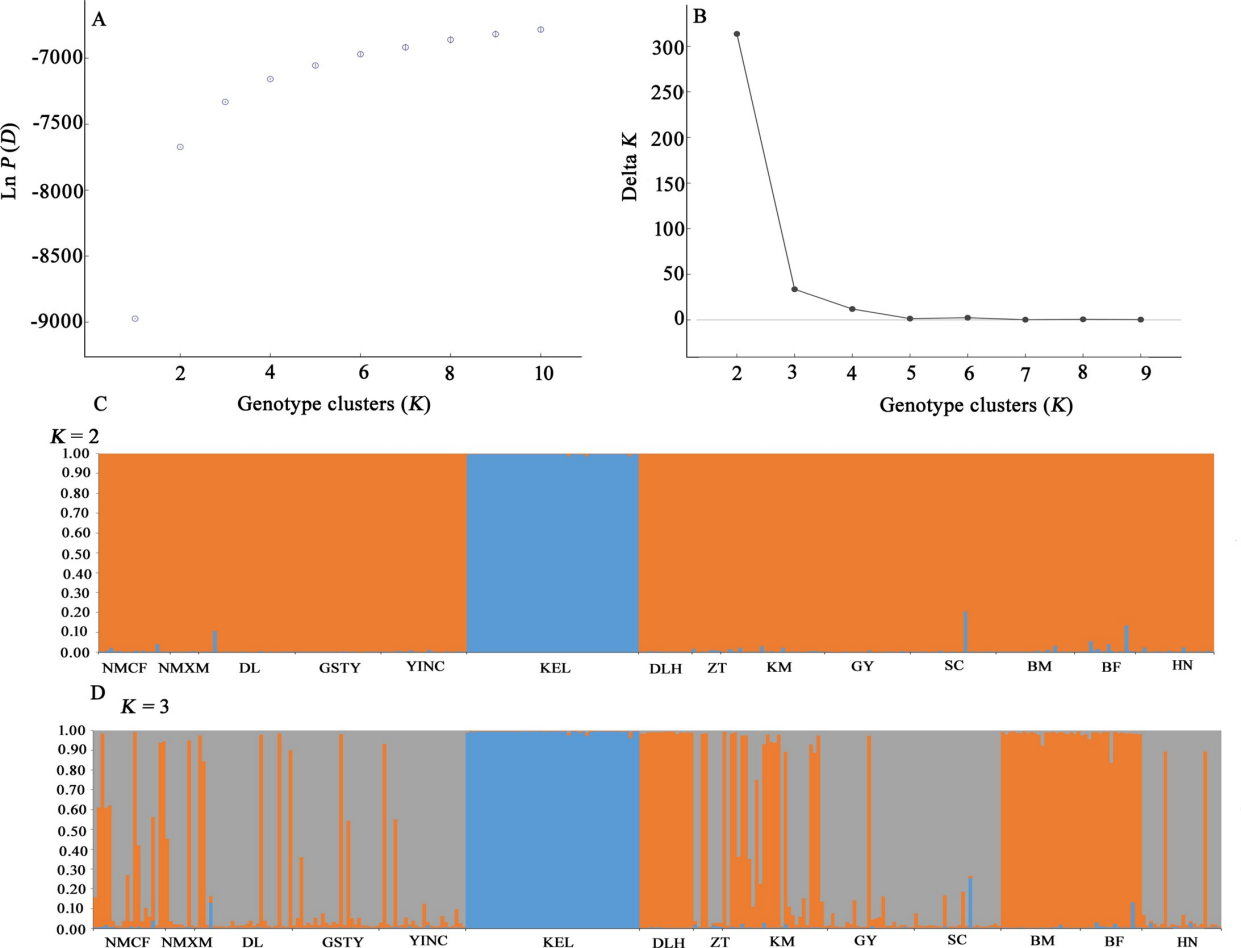
Population structure analysis of 312 individuals collected from 14 *S*. *exigua* populations in western China based on eight microsatellite loci. The likelihood of the data given (a) the mean posterior probability values (the mean ln*P*(*D*) values) and (b) *ΔK* values are plotted against the number of genetic clusters (*K*). (c,d) Individual Bayesian assignment probabilities for *K* = 2 and *K* = 3. Each individual is represented by a single vertical line. The sampling location codes are given in the S1 Table.

Based on microsatellite data, we used a clustering algorithm in STRUCTURE 2.3.3 to infer the relationships between the 14 *S*. *exigua* populations in western China (Fig 3). The mean Ln*P*(*D*) values increased slowly starting from *K* = 2, likely representing the most appropriate number of major clusters. The maximum Δ*K* was reached at *K* = 2. This result was consistent with the hypothesis that these populations could be divided into two groups: the Korla (KEL) group and the remaining group of 13 populations. Further structuring indicated the divergence of Delingha (DLH), Multan (BM), and Faisalabad (BF) from the other populations at *K* = 3. This result was consistent with the NJ phylogenetic tree analyses.

#### Principal coordinate analysis (PCoA)

**Fig 4:**
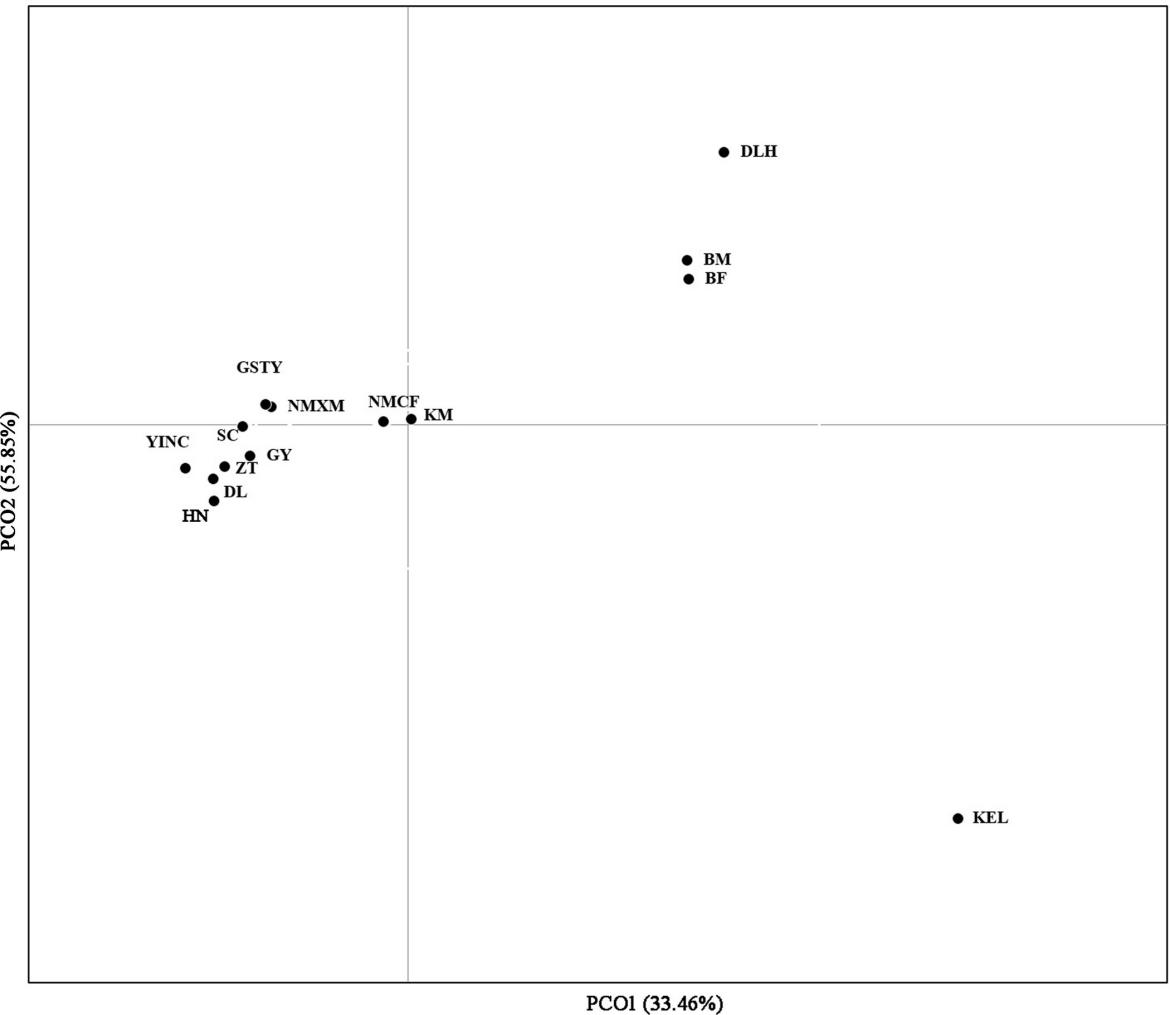
Principal coordinate analysis (PCoA) representing relationships among 14 *S*. *exigua* populations in western China based on the genetic distance matrix of *F*_ST_ values for microsatellite data. Population codes are given in S1 Table.

The population-based PCoA was performed based on a Nei's genetic distance matrix using allele frequencies of the eight microsatellite markers in the 14 *S*. *exigua* populations (Fig 4). The first and second axes account for 33.46% and 55.85% of the total variance, respectively. The above neighbor-joining tree and Bayesian clustering, based on these data collected from 312 individuals, indicated two distinct groups, which is supported by the efficacy of the PCoA approach.

#### Analysis of molecular variance (AMOVA)

The global AMOVA indicated that greater genetic variance was partitioned within individuals (microsatellite data: 78.31%, mtDNA *COI*: 62.46%), and the remaining genetic variation (microsatellite data: 21.69%, mtDNA *COI*: 10.85%) could be explained by the variation among populations (Table 2). The hierarchical analysis demonstrated that only 28.28% and 6.87% of the variation was explained by the variation among two groups based on microsatellite data and mtDNA *COI* data, respectively, whereas the most variation (microsatellite data: 62.46%, mtDNA *COI*: 88.77%) was explained by variation within populations. Consistent with the results of the NJ tree, PCoA and STRUCTURE, the AMOVA analyses were also supported one genetic group.

**Table 2:** Results of molecular variance (AMOVA) test on mitochondrial and microsatellite markers.

Molecular marker	Source of variation	Sum of squares	Variance components	Percentage variation (%)	*F*-statistics
Microsatellite	Global analysis				
	Among populations	154.029	0.249Va	21.69	*F*_ST_ = 0.217***
	Within populations	548.570	0.899 Vb	78.31	
	Total	702.599	1.148	100.00	
	Hierarchical AMOVA (*K* = 2)				
	Among groups	78.793	0.407Va	28.28	*F*_ST_ = 0.375*** F_SC_ = 0.129*** F_CT_ = 0.283
	Among populations within groups	75.237	0.133Vb	9.26	
	Within populations	548.570	0.899 Vc	62.46	
	Total	702.599	1.440	100.00	
mtDNA *COI*	Global analysis				
	Among populations	8.145	0.022 Va	10.85	*F*_ST_ = 0.109***
	Within populations	49.663	0.179 Vb	89.15	
	Total	57.808	0.201 Vc	100.00	
	Hierarchical AMOVA (*K* = 2)				
	Among groups	2.014	0.015 Va	6.87	*F*_SC_ = 0.090*** F_ST_ = 0.152*** F_CT_ = 0.069
	Among populations within groups	6.130	0177 Vb	8.35	
	Within populations	49.663	0.179 Vc	84.77	
	Total	57.808	0.211	100.00	

*d*.*f*., degree of freedom; *P < 0.05,

***P < 0.001: significance level. Two groups including eastern region group and western region group based on STRUCTURE analysis (See Fig 3).

#### Isolation By Distance (IBD)

**Fig 5:**
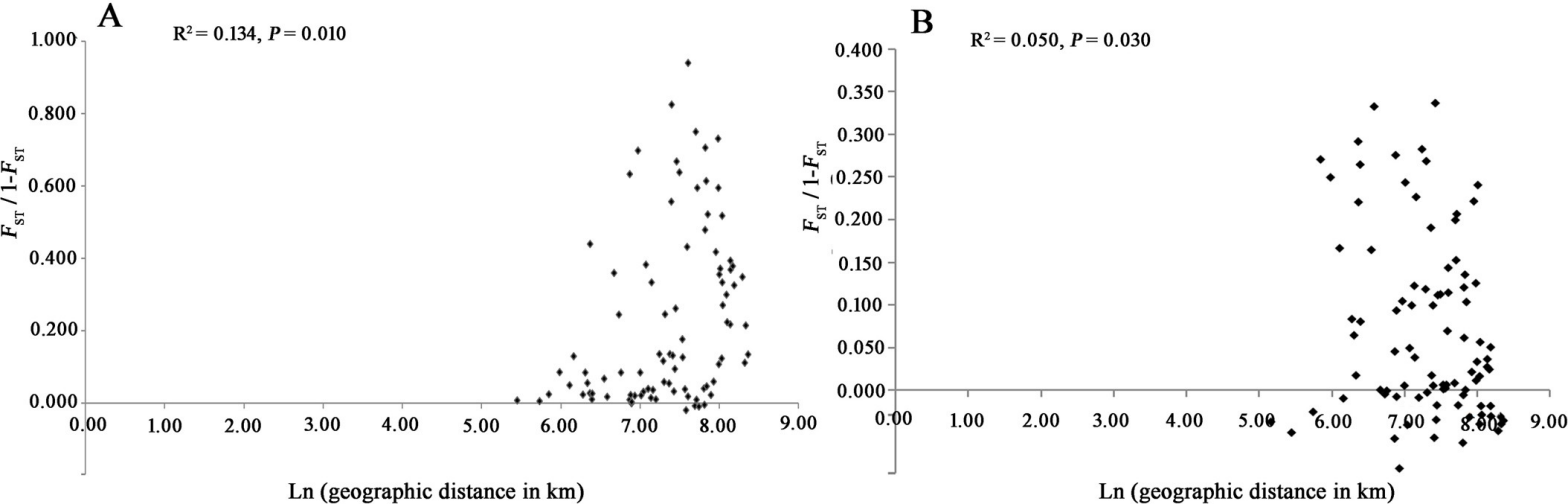
Correlation analysis between the pairwise *F*_ST_/1–*F*_ST_ values and the logarithm of geographic distance based on **(a)** microsatellites and **(b)** mtDNA *COI* sequences.

A Mantel test for IBD was conducted to estimate the correlation between the genetic distance and geographic distance of *S*. *exigua*. If the dispersal of *S*. *exigua* is limited by distance, genetic and geographical distances should be positively correlated, producing a pattern of isolation by distance. Applying the Mantel test, the weak significant positive correlation between genetic and geographic distance was observed among any of the *S*. *exigua* populations in western China (microsatellites: R^2^ = 0.134, *P* = 0.010, Fig 5A; mtDNA: R^2^ = 0.050, *P* = 0.030, Fig 5B). In contrast to the mtDNA data, the microsatellite data were more in line with the IBD pattern, perhaps as a result of the limited mtDNA gene sequence variable information and the relatively slower evolution rate.

#### Demographic history

Based on the mtDNA *COI* data, neutrality tests were conducted using Tajima’s *D* [31] and Fu’s *F*_S_ [32] statistics. Significantly negative values were obtained (Tajima’s *D* = –1.678, *P* < 0.05, Fu’s *F*_S_ = –7.987) (S6 Table). The widespread single haplotype (Hap 2) and the star-like haplotype network map provide strong evidence of expansion (Fig 1). The unimodal mismatch distribution of all populations also indicated a sudden demographic expansion in these populations (S1 Fig).

The bottleneck effect analysis based on the microsatellite data showed no significant heterozygote excess in 13 of the 14 populations (except for the DLH population) under three models (IAM, TPM, and SMM), which is consistent with a normal L-shaped distribution of allelic frequency. Therefore, these results indicated that *S*. *exigua* had expanded demographically without experiencing a severe population bottleneck in western China (S7 Table).

#### Individual assignment and migrant detection

In this study, we detected a high individual assignment success, 191 of 312 individuals (61.0%) were definitively classified at a *P* value of 0.05 based on the partial Bayesian method. Our results showed 130 migrant individuals from all of the 312 individuals, and the majority of putative migrant genotypes (110/135) were restricted to three populations of southwestern China, including Kunming (KM), Guiyang (GY) and Chengdu (SC). It also provides a highly directional from southwest China to other western regions populations. In addition, we also observed 11 individuals as potentially first-generation migrants (F0), of which 5 individuals migrated from three southwestern populations to almost all the other populations of *S*. *exigua* (S8 Table).

## Discussion

### Genetic variation and genetic differentiation

In the present study, *S*. *exigua* was found to have low haplotype diversity and low nucleotide diversity based on the mtDNA *COI* gene (*Hd* = 0.273 ± 0.034, *Pi* = 0.00069 ± 0.00010), and a moderate expected heterozygosity (*He*) of 0.605 based on the microsatellite loci, demonstrating a low level of genetic diversity in *S*. *exigua* in western China. This result is consistent with previous studies [17,20,21]. Similarly, several studies suggested a low level of genetic diversity in other migratory Lepidoptera, such as monarch butterflies (*Danaus plexippus*) [47]. In general, species that are capable of dispersal show little genetic differentiation between populations. *S*. *exigua* is a significant agriculture pest, but little information has been published about its dispersal ability [48]. Overall, the low genetic differentiation among most populations implies that the relatively active dispersal capacity might result in increased gene flow. However, distinct ecological climates in different regions might also contribute to the limitation of gene flow among regions. In the present study, we found a significant genetic differentiation between Korla (KEL) and all remaining populations based on the microsatellite and mitochondrial DNA data. This was caused by the long-term independent diversification of these two lineages, along with other factors such as geographic barriers and temperature limitations, and these factors may play important roles in maintaining the present phylogeographic patterns. This result is consistent with findings regarding the diamondback moth, *Plutella xylostella*, where sampling locations throughout temperate and subtropical Chinese regions were based on the climate division in China [49].

### Population genetic structure

Knowledge of the population genetic structure can provide insight into the evolutionary and ecological processes of species. In the present study, we found no distinct distribution pattern of this pest based on mtDNA haplotypes phylogenetic tree. In line with that, the mtDNA haplotypes network analysis showed that the dominant haplotype (Hap2) was evenly distributed in the western regions of China, indicated that gene flow occurred among the populations of this species in recent history and blurred the original relationship between different populations. These genetic features are evidence of migration, as demonstrated in other migratory species [47,50–52]. POPTREE, PCoA, STRUCTURE, and AMOVA analyses based on microsatellite data divided all individuals into two clusters, the first cluster including the Korla (KEL) population, and the second clade composed of the remaining 13 populations in western China. To the best of our knowledge, geographic barriers and climatic factors might contribute to the isolation of the studied population and they might play an important role in preventing gene flow among groups [53,54]. For example, mountains shaped the population structure of the terrestrial species *Locusta migratoria* [55] and *Paeonia rockii* [56]. We speculate that the differing climates and large mountain systems in western China might have prevented or slowed nuclear gene flow among the western populations of *S*. *exigua*, resulting in lineage differentiation. For example, Mt. Tianshan might have acted as a substantial geographical barrier, producing significant population differentiation between KEL and the remaining populations. Similarly, genetic structuring on a local scale has also been reported in the migratory moth *Helicoverpa armigera* in Australia, where it is thought that drought conditions impacted its migratory capacity and resulting genetic structure [57]. We found weak significant positive correlation between genetic and geographic distance among all of the *S*. *exigua* populations in western China, and this result is consistent with those reported in previous studies [17,21,22]. IBD affects are pronounced in moderately mobile species or are weak in both low-mobility and high-mobility species since extensive gene flow homogenizes populations across the large scale of geographic regions [58].

### Pest management implications

*S*. *exigua* is a polyphagous species that has been described as feeding on more than 300 plants, which implies its considerable adaptive potential. This pest originates from South Asia. It is currently widely distributed in most of the main crop-producing areas in China. We collected five individuals of *S*. *exigua* using sex pheromone traps for the first time in Yichun, Heilongjiang, in 2012. Therefore, this species has not accumulated additional genetic variation due to its short history of expansion in Northern China. In the present study, we found a high level of genetic diversity in the Kunming (KM) population. This high level of diversity can probably be explained by *S*. *exigua* not experiencing serious founder effects, genetic bottlenecks, or strong selection (e.g., from insecticides) in this region. Resistance to insecticides in insects is an example of evolutionary adaptation to environmental changes [59]. Since the past several decades, cultural and chemical controls have been used to prevent the spread and damage of *S*. *exigua*. *S*. *exigua* has developed resistance to some insecticides. For example, this species has a long history of exposure to carbamate pesticides and has developed a high level of resistance to methomyl in California, USA, since 1989 [9]. Therefore, quantifying the level of resistance to insecticides and investigating the distribution of resistance genes relative to the genetic structure and the level of gene flow among Chinese *S*. *exigua* populations are essential. Understanding the dispersal ability, genetic structure, and population demography of this pest is critical for determining both theoretical aspects of its evolution and the effective implementation of pest forecasting systems. In the future, we will explore population genetic differentiation and population genetic structure based on the genomic level of *S*. *exigua*, and deeply reveal the evolution relationship, reconstruct population history, it will help us to gain insight into the adaptation to climates and ecology factors at the genomic level. These works will be carried out before a reasonable control strategy is developed for managing *S*. *exigua*.

## Conclusions

We used mitochondrial DNA (mtDNA) fragments of the *cytochrome oxidase subunit I* (*COI*) and eight nuclear microsatellite loci to investigate genetic diversity and population genetic structure of *S*. *exigua* populations at 14 sampling sites in Western China. Low levels of genetic diversity and a moderate genetic differentiation among *S*. *exigua* populations were detected. Population genetic structure analysis revealed two genetically distinct groups: the KEL group and the remaining population group. Isolation by distance (IBD) results showed no significant correlation between geographic distance and genetic differentiation. Population demographic history analysis indicated that the beet armyworm experienced a recent rapid expansion without a recent genetic bottleneck in western China.

## Supporting information

S1 Fig Analyses of mismatch distributions and the neutrality test results of Tajima’s *D* and Fu’s *F*_S_ tests for sampling localities in the total population of western China based on *COI* sequences. The *x*-axis represents the numbers of pairwise differences; the *y*-axis represents the relative frequency. The expected distribution under a model of population expansion is given as a continuous line, and the observed distribution is given as a dashed line.
(TIF)

S1 Table Habitat location, sampling size and regional group information of *Spodoptera exigua* in different geographic populations of Western China. (DOCX)

S2 Table Estimates of null allele frequency for each locus. (DOCX)

S3 Table Estimates of *F*_ST_ without and with the ENA correction for each locus. (DOCX)

S4 Table Genetic variation and the gene flow among 8 microsatellite loci of *Spodoptera exigua* in western China. (DOCX)

S5 Table List of populations of *Spodoptera exigua* studied indicating the genetic diversity at 8 microsatellite loci. (DOCX)

S6 Table Distribution of the haplotypes, genetic diversity and neutral test among different geographic populations of *Spodoptera exigua* in western China based on mtDNA *COI* sequences. (DOCX)

S7 Table Wilcoxon signed-rank test for mutation–drift equilibrium estimated based on 10 microsatellite loci. (DOCX)

S8 Table Results of assignment test and detection of first generation migrants (F0) based on individuals, with source populations list by column and recipient populations by row. (DOCX)
