# Supplementary figures and images for: Genetic variation and phylogeographic structure of Spodoptera exigua in western China based on mitochondrial DNA and microsatellite markers
Source: PLoS One. 2020 May 14;15(5):e0233133. doi: 10.1371/journal.pone.0233133 (PMC7224464; doi:10.1371/journal.pone.0233133)

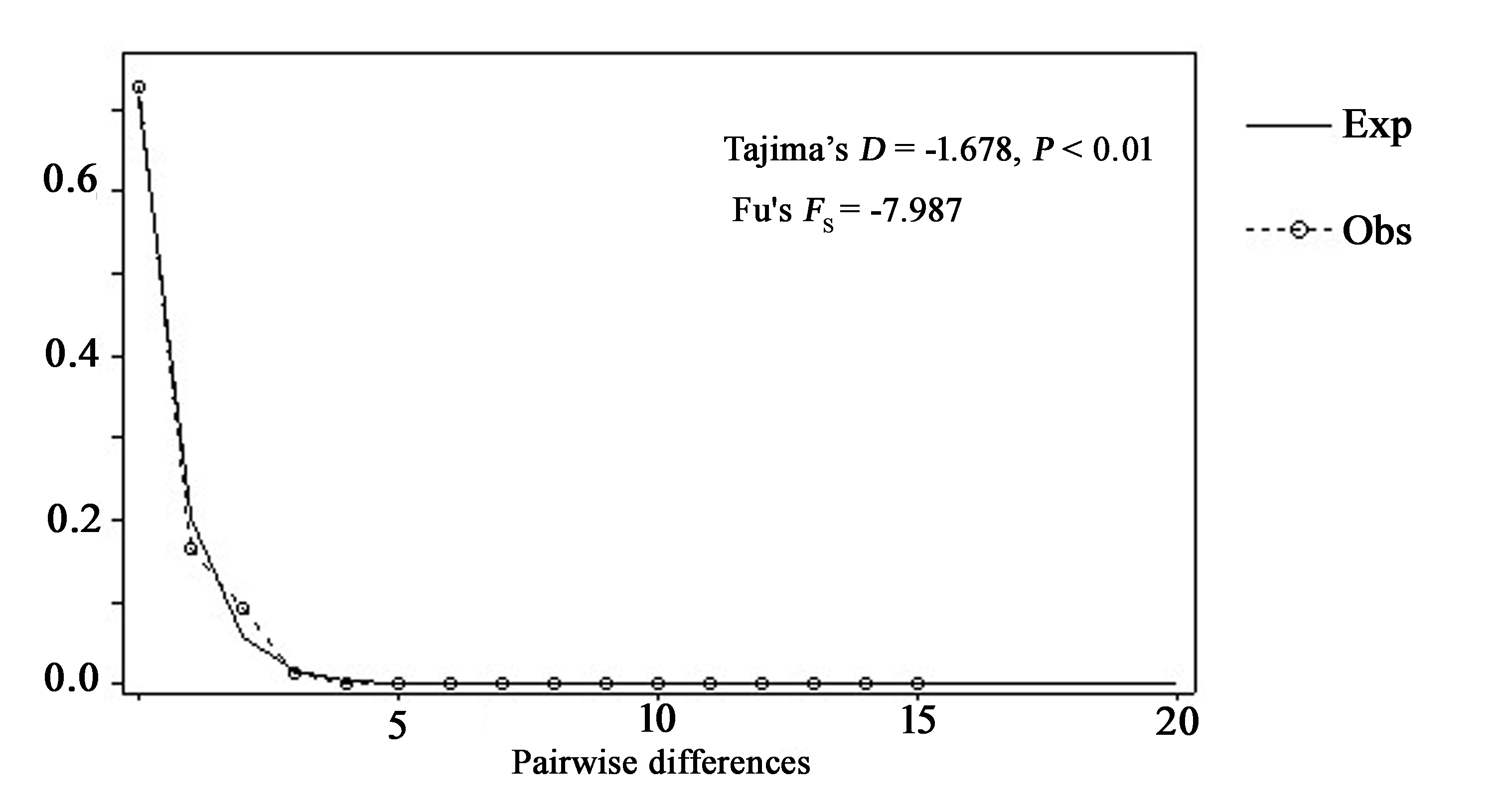

Supplement: S1 Fig — The x-axis represents the numbers of pairwise differences; the y-axis represents the relative frequency. The expected distribution under a model of population expansion is given as a continuous line, and the observed distribution is given as a dashed line. (TIF) [file pone.0233133.s001.tif]
