# Supplementary material for: Genetic variation and phylogeographic structure of Spodoptera exigua in western China based on mitochondrial DNA and microsatellite markers
Source: PLoS One. 2020 May 14;15(5):e0233133. doi: 10.1371/journal.pone.0233133 (PMC7224464; doi:10.1371/journal.pone.0233133)
Supplement: S1 Table — (DOCX) [file pone.0233133.s002.docx]

**S1 Table.** **Sampling location, sampling size and regional group information of *Spodoptera exigua* in different geographic populations of western China**

| **Sampling locations / Zone** | **Site code** | ***COI*** | **SSR** | **Insect stage** | **Latitude / Longitude** | **Collection date** | **Altitude (m asl)** | Host plant |
| --- | --- | --- | --- | --- | --- | --- | --- | --- |
| Chifeng, Inner Mongolia/ MTZ | NMCF | 16 | 20 | Adult | 43.94 °N, 117.88 °E | January 2018 | 1040 | Welsh onion |
| Xilengrad, Inner Mongolia/ MTZ | NMXM | 8 | 8 | Adult | 42.08 °N, 116.56 °E | July 2017 | 1957 | Corn |
| Dali, Shaaxi / WTZ | DL | 22 | 27 | Adult | 34.79 °N, 110.10 °E | August 2012 | 334 | Welsh onion |
| Anning, Lanzhou, Gansu/ MTZ | GSTY | 24 | 24 | Adult | 36.10 °N, 103.69 °E | July 2017 | 1531 | Corn |
| Yinchuan, Xining/ MTZ | YINC | 21 | 24 | Adult | 38.47 °N, 106.25 °E | July 2017 | 1010 | Corn |
| Korla, Xinjiang/ WTZ | KEL | 45 | 48 | Adult | 41.73 °N, 87.17 °E | June 2017 | 934 | Corn |
| Delingha, Qinghai/ QTPZ | DLH | 15 | 15 | Adult | 36.55 °N, 96.15 °E | August 2012 | 2982 | Corn |
| Zhaotong, Yunnan / SZ | ZT | 8 | 8 | Adult | 27.33 °N, 103.73 °E | August 2012 | 1905 | Corn |
| Kunming, Yunnan / SZ | KM | 29 | 29 | Adult | 23.58 °N, 102.01 °E | August 2012 | 1930 | [Chinese cabbage](http://www.baidu.com/link?url=iCHd1pbh6SDUkZjvBIrcG1WRAzobiLGREyotBNj9HKipAX3LFTbscl9UkAk_fs40N11gVosjEiGOeMRIAPkvSiDtXQteEhwCBXQvBswitVlJ5Q6DvA6fLgaSMNqOQnAH) |
| Huaxi, Guiyang, Guizhou/ SZ | GY | 24 | 24 | Adult | 26.41 °N, 106.67 °E | September 2017 | 1762 | Corn |
| Chengdu, Sichuan/ SZ | SC | 20 | 24 | Adult | 30.88 °N, 104.25 °E | September 2017 | 475 | Corn |
| Multan, Pakistan | BM | 22 | 22 | Adult | 31.08 °N, 71.30 °E | July 2018 | 500 | Cotton |
| Faisalabad, Pakistan | BF | 15 | 17 | Adult | 31.39 °N, 73.03 °E | June 2018 | 185 | Cotton |
| Hanoi, Vietnam | HN | 22 | 22 | Adult | 21.07 °N, 105.75 °E | June 2018 | 15.7 | [Chinese cabbage](http://www.baidu.com/link?url=iCHd1pbh6SDUkZjvBIrcG1WRAzobiLGREyotBNj9HKipAX3LFTbscl9UkAk_fs40N11gVosjEiGOeMRIAPkvSiDtXQteEhwCBXQvBswitVlJ5Q6DvA6fLgaSMNqOQnAH) |

Sites are labeled according to abbreviated location names for *Spodoptera exigua,* MTZ, mid-temperature zone; WTZ, warm temperate zone; TZ, tropical zone; SZ, subtropical zone; QTPZ, Qinghai-Tibet plateau zone.
