# Supplementary material for: Genetic variation and phylogeographic structure of Spodoptera exigua in western China based on mitochondrial DNA and microsatellite markers
Source: PLoS One. 2020 May 14;15(5):e0233133. doi: 10.1371/journal.pone.0233133 (PMC7224464; doi:10.1371/journal.pone.0233133)
Supplement: S2 Table — (DOCX) [file pone.0233133.s003.docx]

**S2 Table. Estimates of null allele frequency for each locus**

| Locus | Null allele frequency |
| --- | --- |
| 1 | 0.012 |
| 2 | 0.099 |
| 3 | 0.067 |
| 4 | 0.134 |
| 5 | 0.046 |
| 6 | 0.108 |
| 7 | 0.076 |
| 8 | 0.099 |
