# Supplementary material for: Genetic variation and phylogeographic structure of Spodoptera exigua in western China based on mitochondrial DNA and microsatellite markers
Source: PLoS One. 2020 May 14;15(5):e0233133. doi: 10.1371/journal.pone.0233133 (PMC7224464; doi:10.1371/journal.pone.0233133)
Supplement: S3 Table — (DOCX) [file pone.0233133.s004.docx]

**S3 Table.** **Estimates of *F*_ST_ without and with the ENA correction for each locus**

| Locus |  | *F*_ST_ not using ENA | *F*_ST_ using ENA |
| --- | --- | --- | --- |
| 1 |  | 0.188 | 0.187 |
| 2 |  | 0.071 | 0.072 |
| 3 |  | 0.197 | 0.181 |
| 4 |  | 0.172 | 0.140 |
| 5 |  | 0.077 | 0.071 |
| 6 |  | 0.432 | 0.414 |
| 7 |  | 0.177 | 0.164 |
| 8 |  | 0.159 | 0.143 |
| Average |  | 0.184 | 0.171 |
| All loci |  | 0.182 | 0.168 |

The excluding null alleles (ENA).
