# Supplementary material for: Genetic variation and phylogeographic structure of Spodoptera exigua in western China based on mitochondrial DNA and microsatellite markers
Source: PLoS One. 2020 May 14;15(5):e0233133. doi: 10.1371/journal.pone.0233133 (PMC7224464; doi:10.1371/journal.pone.0233133)
Supplement: S4 Table — (DOCX) [file pone.0233133.s005.docx]

**S4 Table. Genetic variation and the gene flow among 8 microsatellite loci of *Spodoptera exigua* in western China**

| Loci | *Na* | *Ne* | *I* | *Ho* | *He* | *uHe* | *F*_ST_ | *F*_IT_ | *F*_IS_ | *Nm* | *Hs* | *A_R_* | *r* |
| --- | --- | --- | --- | --- | --- | --- | --- | --- | --- | --- | --- | --- | --- |
| spe06 | 3.000 | 2.042 | 0.791 | 0.666 | 0.476 | 0.529 | 0.328 | 0.060 | -0.400 | 0.512 | 0.645 | 7.000 | 0.012 |
| spe11 | 2.375 | 1.307 | 0.568 | 0.337 | 0.314 | 0.326 | 0.646 | 0.621 | -0.073 | 0.137 | 0.650 | 7.976 | 0.099 |
| spe08 | 7.563 | 4.350 | 1.600 | 0.708 | 0.726 | 0.762 | 0.142 | 0.164 | 0.026 | 1.506 | 0.846 | 14.672 | 0.067 |
| spe09 | 8.188 | 4.607 | 1.629 | 0.575 | 0.726 | 0.761 | 0.187 | 0.356 | 0.208 | 1.086 | 0.889 | 27.735 | 0.134 |
| spe15 | 8.313 | 5.590 | 1.780 | 0.813 | 0.776 | 0.815 | 0.156 | 0.116 | -0.048 | 1.349 | 0.901 | 20.794 | 0.046 |
| spe07 | 3.313 | 1.362 | 0.531 | 0.190 | 0.251 | 0.289 | 0.684 | 0.760 | 0.241 | 0.116 | 0.692 | 23.525 | 0.108 |
| spe10 | 5.125 | 2.316 | 1.003 | 0.414 | 0.476 | 0.496 | 0.459 | 0.530 | 0.131 | 0.294 | 0.568 | 15.837 | 0.076 |
| spe12 | 5.500 | 2.752 | 1.100 | 0.441 | 0.519 | 0.541 | 0.396 | 0.487 | 0.150 | 0.381 | 0.835 | 20.837 | 0.099 |
| Overall | 5.422 | 3.041 | 1.125 | 0.518 | 0.533 | 0.565 | 0.375 | 0.387 | 0.029 | 0.673 | 0.753 | 17.297 | 0.080 |

Abbreviations: *Na*, Observed number of alleles; *Ne*, Effective number of alleles; *I*, Shannon's information index; *Ho*, Observed heterozygosity; *He*, Expected heterozygosity; *uHe*, Unbiased expected heterozygosity; *F*_ST_, Fixation index; *F*_IT_, Total population inbreeding coefficient; *F*_IS_, Inbreeding coefficient; *Nm*, Gene flow; *H*_S_, gene diversity; *A_R_*, Allelic Richness; *r*, frequency of null alleles.
