# Supplementary material for: Genetic variation and phylogeographic structure of Spodoptera exigua in western China based on mitochondrial DNA and microsatellite markers
Source: PLoS One. 2020 May 14;15(5):e0233133. doi: 10.1371/journal.pone.0233133 (PMC7224464; doi:10.1371/journal.pone.0233133)
Supplement: S5 Table — (DOCX) [file pone.0233133.s006.docx]

**S5 Table. List of populations of *Spodoptera exigua* studied indicating the genetic diversity at 8 microsatellite loci**

| Pop | *N*_a_ | *N*_e_ | *I* | *H*_o_ | *H*_e_ | *uH*_E_ | *F* | *F*_IS_ | *H*_S_ | *A*_R_ | *A*_P_ |
| --- | --- | --- | --- | --- | --- | --- | --- | --- | --- | --- | --- |
| NMCF | 6.125 | 3.947 | 1.366 | 0.601 | 0.644 | 0.663 | 0.052 | 0.098 | 0.665 | 4.121 | 0.125 |
| NMXM | 4.250 | 3.007 | 1.099 | 0.493 | 0.563 | 0.607 | 0.175 | 0.192 | 0.617 | 3.686 | 0.000 |
| DL | 7.375 | 3.751 | 1.463 | 0.500 | 0.664 | 0.677 | 0.315 | 0.265 | 0.681 | 4.257 | 0.250 |
| GSTY | 5.125 | 3.221 | 1.173 | 0.547 | 0.587 | 0.600 | 0.132 | 0.095 | 0.601 | 3.484 | 0.125 |
| YINC | 7.125 | 3.817 | 1.358 | 0.496 | 0.611 | 0.625 | 0.195 | 0.200 | 0.629 | 4.062 | 0.250 |
| KEL | 6.250 | 2.957 | 1.156 | 0.442 | 0.547 | 0.553 | 0.249 | 0.206 | 0.555 | 3.438 | 3.250 |
| DLH | 4.250 | 2.908 | 1.016 | 0.469 | 0.527 | 0.546 | 0.156 | 0.133 | 0.549 | 3.219 | 0.125 |
| ZT | 4.500 | 3.115 | 1.147 | 0.478 | 0.576 | 0.622 | 0.190 | 0.217 | 0.636 | 3.944 | 0.000 |
| KM | 8.875 | 3.872 | 1.545 | 0.588 | 0.681 | 0.693 | 0.135 | 0.157 | 0.695 | 4.414 | 0.750 |
| GY | 7.500 | 4.166 | 1.507 | 0.639 | 0.666 | 0.681 | 0.069 | 0.066 | 0.682 | 4.472 | 0.375 |
| SC | 5.500 | 2.935 | 1.136 | 0.654 | 0.569 | 0.583 | -0.191 | -0.090 | 0.580 | 3.360 | 0.125 |
| BM | 6.750 | 3.682 | 1.383 | 0.665 | 0.658 | 0.674 | -0.053 | 0.010 | 0.678 | 4.036 | 0.375 |
| BF | 6.375 | 3.774 | 1.399 | 0.606 | 0.664 | 0.690 | 0.036 | 0.116 | 0.694 | 4.250 | 0.375 |
| HN | 4.875 | 3.655 | 1.130 | 0.443 | 0.517 | 0.529 | 0.103 | 0.166 | 0.531 | 3.638 | 0.000 |
| Average | 6.063 | 3.486 | 1.277 | 0.544 | 0.605 | 0.624 | 0.114 | 0.131 | 0.628 | 3.884 | 0.438 |

Abbreviations: *N*_a_, observed number of alleles; *N*_e_*,* effective number of alleles; *I*, Shannon’s information index, *H*_o_, observed heterozygosity, *H*_e_, expected heterozygosity; *uH*_E_, unbiased expected heterozygosity; *F*, fixation index; *F*_IS_, inbreeding index; *H*_S_, gene diversity; *A*_R_, allelic richness; *A*_P_, number of private alleles.
