# Supplementary material for: Genetic variation and phylogeographic structure of Spodoptera exigua in western China based on mitochondrial DNA and microsatellite markers
Source: PLoS One. 2020 May 14;15(5):e0233133. doi: 10.1371/journal.pone.0233133 (PMC7224464; doi:10.1371/journal.pone.0233133)
Supplement: S6 Table — (DOCX) [file pone.0233133.s007.docx]

**S6 Table.** **Distribution of the haplotypes, genetic diversity and neutral test among different geographic populations of *Spodoptera exigua* in western China based on mtDNA *COI* sequences**

| Locality | Haplotype | N / H | *h* ± SD | *π* ± SD | *k* | Tajima’s *D* | Fu's *F_S_* |
| --- | --- | --- | --- | --- | --- | --- | --- |
| NMCF | Hap_1(1) \ Hap_2(15) | 16 / 2 | 0.125 ± 0.106 | 0.00022 ± 0.00018 | 0.12500 | -1.16221 | -0.700 |
| NMXM | Hap_2(8) | 8 / 1 | 0.000 ± 0.000 | 0.00000 ± 0.00000 | 0.00000 | 0.00000 | 0.000 |
| DL | Hap_3(5) \ Hap_2(17) | 22 / 2 | 0.368 ± 0.100 | 0.00128 ± 0.00035 | 0.73593 | 0.77341 | 2.259 |
| GSTY | Hap_4(9) \ Hap_2(15) | 24 / 2 | 0.489 ± 0.057 | 0.00085 ± 0.00010 | 0.48913 | 1.39118 | 1.462 |
| YINC | Hap_3(1) \ Hap_2(20) | 21 / 2 | 0.095 ± 0.084 | 0.00033 ± 0.00029 | 0.19048 | -1.51414 | -0.070 |
| KEL | Hap_6(1) \ Hap_5(1) \ Hap_4(11) \Hap_2(32) | 45 / 4 | 0.443 ± 0.070 | 0.00089 ± 0.00019 | 0.51111 | -1.02498 | -0.854 |
| DLH | Hap_3(1) \ Hap_5(2) \ Hap_2(12) | 15 / 3 | 0.362 ± 0.145 | 0.00089 ± 0.00043 | 0.51429 | -1.31654 | -0.379 |
| ZT | Hap_3(2) \ Hap_2(6) | 8 / 2 | 0.429 ± 0.169 | 0.00149 ± 0.00059 | 0.85714 | 0.41421 | 1.653 |
| KM | Hap_3(1) \ Hap_7(1) \ Hap_2(27) | 29 / 3 | 0.135 ± 0.085 | 0.00036 ± 0.00024 | 0.20690 | -1.73263 | -1.585 |
| GY | Hap_3(3) \ Hap_2(21) | 24 / 2 | 0.228 ± 0.102 | 0.00079 ± 0.00035 | 0.45652 | -0.32459 | 1.333 |
| SC | Hap_8(1) \ Hap_2(19) | 20 / 2 | 0.100 ± 0.088 | 0.00017 ± 0.00015 | 0.10000 | -1.16439 | -0.879 |
| BM | Hap_1(1) \ Hap_3(1) \ Hap_9(1) \ Hap_2(19) | 22 / 4 | 0.260 ± 0.120 | 0.00062 ± 0.00031 | 0.35498 | -1.47087 | -2.262 |
| BF | Hap_10(1) \ Hap_2(14) | 15 / 2 | 0.133 ± 0.112 | 0.00069 ± 0.00058 | 0.40000 | -1.68500 | 0.834 |
| HN | Hap_2(22) | 22 / 1 | 0.000 ± 0.000 | 0.00000 ± 0.00000 | 0.00000 | 0.00000 | 0.000 |
| Total | Hap_1-Hap_10 (291) | 291 / 10 | 0.273 ± 0.034 | 0.00069 ± 0.00010 | 0.39867 | -1.678^**^ | -7.987 |

N, sample size; H, number of haplotypes; *h*, haplotype diversity; *π*, nucleotide diversity; haplotype names in boldface indicate shared haplotypes; SD, standard deviation; K: average number of nucleotide differences; ^*^*P* < 0.05, ^**^*P* < 0.01, ^***^*P* < 0.001.
