# Supplementary material for: Genetic variation and phylogeographic structure of Spodoptera exigua in western China based on mitochondrial DNA and microsatellite markers
Source: PLoS One. 2020 May 14;15(5):e0233133. doi: 10.1371/journal.pone.0233133 (PMC7224464; doi:10.1371/journal.pone.0233133)
Supplement: S7 Table — (DOCX) [file pone.0233133.s008.docx]

**S7 Table.** **Wilcoxon signed rank test for mutation–drift equilibrium estimated based on 10 microsatellite loci**

| Pop. | IAM | TPM | SMM | Mode shift |
| --- | --- | --- | --- | --- |
| NMCF | 0.037 | 0.230 | 0.727 | L |
| NMXM | 0.273 | 0.629 | 0.727 | L |
| DL | 0.320 | 0.770 | 0.994 | L |
| GSTY | 0.191 | 0.422 | 0.629 | L |
| YINC | 0.527 | 0.844 | 0.980 | L |
| KEL | 0.422 | 0.875 | 0.980 | L |
| DLH | **0.020** | 0.230 | 0.422 | S |
| ZT | 0.527 | 0.727 | 0.973 | L |
| KM | 0.727 | 0.986 | 0.996 | L |
| GY | 0.156 | 0.422 | 1.000 | L |
| SC | 0.055 | 0.711 | 0.988 | L |
| BM | 0.125 | 0.680 | 0.973 | L |
| BF | 0.230 | 0.680 | 0.844 | L |
| HN | **0.008** | **0.016** | **0.039** | L |
| Total region | 0.371 | 0.963 | 1.000 | L |

*P* is test for heterozygosity excess, bold indicates significant at *P* = 0.05; Pop.: population; IAM: infinite allele model; TPM: two-phase model; SMM: stepwise mutation model; L: normal L-shaped distribution; S: shifted mode; *P* is test for heterozygosity deficiencies.
