# Supplementary material for: Genetic variation and phylogeographic structure of Spodoptera exigua in western China based on mitochondrial DNA and microsatellite markers
Source: PLoS One. 2020 May 14;15(5):e0233133. doi: 10.1371/journal.pone.0233133 (PMC7224464; doi:10.1371/journal.pone.0233133)
Supplement: S8 Table — (DOCX) [file pone.0233133.s009.docx]

**S8 Table.** **Results of assignment test and detection of the first generation migrants (F0) based on individuals, with source populations list by column and recipient populations by row**

|  | NMCF | NMXM | DL | GSTY | YINC | KEL | DLH | ZT | KM | GY | SC | BM | BF | HN |
| --- | --- | --- | --- | --- | --- | --- | --- | --- | --- | --- | --- | --- | --- | --- |
| NMCF | 6 | 5 | 1 | 4 |  |  |  |  |  |  |  | 1 | 1 | 2 |
| NMXM | 3 | 1 | 1 | 1 |  |  |  | 1 |  |  | 1 |  |  |  |
| DL | 1 | 1 | 14 (1) |  | 3 |  |  | 3 | 1 | 3 |  |  |  | 1 |
| GSTY | (1) | 1 | 1 | 16 |  |  |  |  |  |  | 4 |  | 1 | 1 |
| YINC | 1 | 5 | 2 (1) | 1 | 7 |  |  |  |  | 6 | 1 |  |  | 1 |
| KEL |  |  |  |  |  | 48 (1) |  |  |  |  |  |  |  |  |
| DLH |  |  |  |  |  |  | 13 |  |  |  |  |  | 2 |  |
| ZT |  | 1 | 1 |  | 1 |  |  | 2 | 2 |  |  |  |  | 1 (1) |
| KM | 1 | 2 | 2 (1) |  | 3 |  |  | 5 | 13 | 2 (1) |  |  |  | 1 |
| GY | 1 | 2 | 3 |  | 6 |  |  | 1 |  | 10 | 1 |  |  |  |
| SC | (1) | 2 |  | 3 |  |  |  | 1 |  |  | 18 (1) |  |  |  |
| BM |  |  |  |  |  |  | 1 |  |  |  |  | 18 | 3 (2) |  |
| BF |  | 1 |  |  |  |  | 3 |  | 9 |  |  | 4 | 9 |  |
| HN |  | 2 |  | 2 |  |  |  | 2 |  |  |  |  |  | 16 |
